# Paradoxical Effect of Aspirin

**DOI:** 10.1155/2012/676237

**Published:** 2012-01-15

**Authors:** Christian Doutremepuich, Omar Aguejouf, Vanessa Desplat, Francisco X. Eizayaga

**Affiliations:** ^1^Laboratoire d'Hématologie, Université Bordeaux Segalen, 146 rue Léo Saignat, 33076 Bordeaux Cedex, France; ^2^CEBBAD, Universidad Maimónides, Buenos Aires C1405BCK, Argentina

## Abstract

Low-dose aspirin is an important therapeutic option in the secondary prevention of myocardial infarction (MI) and ischemic stroke, basedon its unique cost-effectiveness and widespread availability. In addition, based on the results of a number of large studies, aspirin is also widely used in the primary prevention of MI. This paper provides an update of the available data to offer greater clarity regarding the risks of aspirin with respect to hemorrhagic stroke. In the secondary prevention of cardiovascular, cerebrovascular, and ischemic events, the evidence supports that the benefits of aspirin treatment significantly outweigh the risk of a major hemorrhage. When considering whether aspirin is appropriate, the absolute therapeutic cardiovascular benefits of aspirin must be balanced with the possible risks associated with its use, being hemorrhagic stroke. Regarding these clinical facts, normal, COX 1 −/−, and COX 2 −/− mice were treated with a wide range of doses of aspirin and studied by induced hemorrhagic time. The results outlined three major conclusions: high doses of aspirin induce hemorrhage, while low doses of aspirin do not. In the absence of COX 1, ultra low doses of aspirin produce an antihemorrhagic effect not observed with intermediate doses. The absence of COX 2 induced a hemorrhagic effect that needs further research, probably originated in compensatory phenomena.

## 1. Introduction

Despite more than 100 years of use, acetyl salicylic acid (aspirin) has only been recognized for the prevention of myocardial infarction (MI) and ischemic stroke for the past 25 years. Over this period, based on its unique cost-effectiveness and widespread availability, the utilization of aspirin has expanded substantially for both primary and secondary prevention of cardiovascular events, providing significant insight into its safety and effectiveness.

The decision as to which patients to treat must weigh the benefits of chronic aspirin therapy against the possible risks associated with its use, including the risk of intracerebral and subarachnoid hemorrhage, the most serious risks associated with the use of aspirin [1–7].

As the number of studies evaluating the long-term use of aspirin has expanded, it is now possible to evaluate the evidence in aggregate to more conclusively estimate the risk of hemorrhagic stroke, allowing a more informative benefit-risk assessment.

The antithrombotic effectiveness of aspirin is related to its inhibition of the cyclooxygenase (COX) enzyme that metabolizes arachidonic acid to a variety of prostanoids, including thromboxane A2 [8]. Platelet-derived cyclooxygenase-1 (COX-1) generates thromboxane A2, a potent vasoconstrictor and platelet agonist. The effect of aspirin on platelet COX-1 is irreversible, thus providing for once-daily low-dose effectiveness. With the inhibition of platelet COX-1 activity, there is a decrease in platelet aggregation, leading to a reduced thromboembolic potential and a commensurate prolonged bleeding time. Thus, it is not surprising that the major risks associated with aspirin relate to bleeding complications.

On the other hand, aspirin treatment must face resistance or variability in response as the cause of treatment failure, which may be due to different kinds of causes. A previous publication of our laboratory has shown that ultra low dose of aspirin induced a prothrombotic effect in rats [9]. This effect of ultra low dose may induce the complications observed after aspirin discontinuation [10].

To study these events, we hypothesized that modifications in the response to aspirin treatment could be due to different doses and an altered response to Cox. We designed an experiment using 72 normal mice and 72 genetically modified male homozygous mice without COX 1 (COX 1 −/−) and 72 lacking COX 2 (COX 2 −/−), where we studied Induced Hemorrhage Time (IHT).

Aspirin was used in a wide spectrum of doses including doses 100 mg/kg/bw and 1 mg/kg/bw and aspirin 1/100 dilutions number 5 (Dil 5), 9 (Dil 9), and 15 (Dil 15), which were obtained by successive 1/100 dilution. Sterilized water was used as placebo. All drugs were injected subcutaneously at a final volume of 1 mL/kg/BW.

## 2. Material and Methods

### 2.1. Animals

Normal mice from centre d'élevage (Depre Saint Doulchard France) and the male homozygous COX 1 −/− and COX 2 −/− mice purchased from Taconic Farms Inc. (Hudson City Centre, NY, USA), were housed separately under conditions of controlled temperature and illumination. They were fed with standard mouse chow and water *ad libitum*. Animals received care in compliance with the European Convention of Animal Care.

### 2.2. Induced Hemorrhagic Time

IHT was performed 10 minutes before thrombosis induction by laser. The tail of the mouse was immersed in water for 5 minutes at 37°C and sectioned 6 mm from the extremity, and the IHT is expressed as the time between the tail section and the end of bleeding, expressed in seconds.

### 2.3. Drugs Tested

The amounts of 1 mg/mL and 100 mg/mL are obtained by dilution of a solution of Acetylsalicylate (Aspegic, Sanofi-synthelabo, France). Aspirin dilutions were prepared as follows: 1 g of pure, finely powdered aspirin was suspended in 99 mL of alcohol (70°). After being vigorously shaken, 1 mL of this dilution was then mixed with 99 mL of distilled water and vigorously shaken (dilution 1). The last process was repeated until obtaining desired dilutions: 4 times (dilution 5), 8 times (dilution 9), and 14 times (dilution 15). Sterilized water (placebo) or aspirin was subcutaneously administered at a final volume of 1 mL/kg mouse weight. The groups were treated with placebo or aspirin in 100 mg/kg or 1 mg/kg, or dilutions 5, 9, or 15. Aspirin or the corresponding placebo was subcutaneously administered at a final volume of 1 mL/kg rat weight.

### 2.4. Distribution of Groups

Normal mice and COX 1 −/− or COX 2 −/− mice were distributed in 6 groups (*n* = 12/group), respectively,

group 1: placebo (sterilized water),

group 2: aspirin 100 mg/kg,

group 3: aspirin 1 mg/kg,

group 4: aspirin dilution 5 (Dil 5),

group 5: aspirin dilution 9 (Dil 9),

group 6: aspirin dilution 15 (Dil 15).

### 2.5. Statistical Analysis

Data are expressed as mean ± SD and compared using one-way analysis of variance (ANOVA) followed by Dunnet's multiple comparison test. A value of *P* < 0.05 was considered as significant. Statistical calculations were performed using Graph Pad Prism version 4.00 for Windows (http://www.graphpad.com). 

## 3. Results

### 3.1. Effects of Aspirin on the Induced Hemorrhagic (IHT) in Normal Mice (Figure 1)

Compared to the control group (113.9 ± 24.64 sec), aspirin administered at 100 mg/kg increases in a statistically significant manner the THP (363.3 ± 93.3 sec). The other amounts of aspirin tested (1 mg/kg, Dil 5, Dil 9 and Dil 15) do not modify the IHT compared to the control group (*P* > 0.05).

### 3.2. Effects of Aspirin on the Induced Hemorrhagic (IHT) in COX 1 −/− Mice (Figure 2)

COX 1 −/− mice control group show an increase in the IHT (255.7 ± 92.3 sec). The amounts of 100 mg/kg, 1 mg/kg, Dil 5, and Dil 9 of Aspirin do not change significantly this effect (288.3 ± 77.0 sec, 287.8 ± 79.2 sec, 275.0 ± 107.2 sec, and 229.3 ± 80.63 sec, resp.).

Administered at Dil 15, Aspirin decreases in a statistically significant manner the IHT (164.8 ± 88.96 sec).

### 3.3. Effects of Aspirin on the Induced Hemorrhagic (IHT) in COX 2 −/− Mice (Figure 3)

COX 2 −/− mice control group show an increase in the IHT (327.3 ± 103.8 sec). In an undifferentiated way, the amounts of 100 mg/kg, 1 mg/kg, Dil 5, Dil 9, and Dil 15 of aspirin do not change significantly this effect (212.2 ± 109.3 sec, 288.1 ± 98.7 sec, 300.9 ± 131.4 sec, 336.5 ± 77.7 sec, and 245.5 ± 123.9 sec, resp.).

## 4. Discussion

Aspirin induces an irreversible inhibition of COX 1, with a subsequent decrease in TXA2 production, in the platelet. This effect is responsible for aspirin's antiaggregant properties which last for approximately 10 days, the lifespan of platelets [8]. Aspirin also inhibits COX 2 but at higher doses than required for COX 1 inhibition [11]. Indeed, it has been reported that 100 mg of aspirin is required to abolish the production of TXA2 in normal individuals [12].

Results from this study showed a statistically significant prolongation in IHT in COX 1 −/− and COX 2 −/− mice, when compared to normal mice.

If the increase in the IHT in mice COX 1 −/− can be explained by the absence of thromboxane A2, confirming its role in the hemorrhagic events, the increase in the IHT in COX 2 −/− mice imposes more research.

The administration of aspirin at 100 mg/kg significantly increases the IHT in the normal mice. In fact, suppression of platelet aggregation and prolongation of bleeding time in presence of ASA at high doses is related to the inhibition of metabolism of arachidonic acid to thromboxane A2, due to the irreversible acetylation of the platelet enzyme cyclooxygenase, when the inhibition of vascular cyclooxygenase leads to the loss of the protective effect of prostacyclin.

The effect of aspirin 100 mg/kg on IHT produced no change when compared to placebo in COX 1 −/− and COX 2 −/− mice. The absence of COX 1 or administration of aspirin at 100 mg/kg had the same effect, and these effects act not in a non synergistic way, as both are supposed to work through a similar mechanism over platelets activities.

Explanations for this effect could include compensation in COX 1 and 2 activity, interaction between COX and NO synthase as suggested by Skill et al. [13], or an effect of aspirin outside the mechanism of COX inhibition like increased NO synthesis by the endothelial cell as suggested by Taubert et al. [14].

Results from this study also demonstrated that the administration of aspirin at 1 mg/kg does not modify the IHT in the normal mice. In the same way, this amount of aspirin does not cause more increase in the IHT observed in COX 1 −/− and COX 2 −/− mice control groups. However, in previous studies, 1 mg/kg of aspirin administered to the rat decreased the formation of thrombi in a significant way. In the normal mice, the administration of aspirin at the Dil 5 and Dil 9 does not modify, compared to the control, the IHT. In the same way, these amounts of aspirin do not modify the increase in the IHT observed in COX 1 −/− and COX 2 −/− mice control groups.

In the normal mice, aspirin at the Dil 15 does not modify the IHT compared to the control group. But, the administration of this same high dilution of aspirin shortened in a statistically significant way the increase of the IHT, observed in the control group of COX 1 −/− mice. This inhibiting effect of Dil 15 is not significant in COX 2 −/− mice.

The above-cited results clearly show that the ultra low doses of aspirin studied have a statistically significant hemorrhagic inhibitory effect. This effect is achieved despite the absence of COX 1, suggesting a different mechanism of action.

In conclusion, this study confirms the hemorrhagic action of aspirin at 100 mg/kg in the absence of COX 1, and antihemorrhagic effect was observed, when administered at dilution 15.

This modifying effect is very marked in the normal mice and in COX 1 −/− mice, in other words in the presence of the COX 2. The hemorrhagic effect observed in COX 2 −/− mice warrants further research.

## Figures and Tables

**Figure 1:**
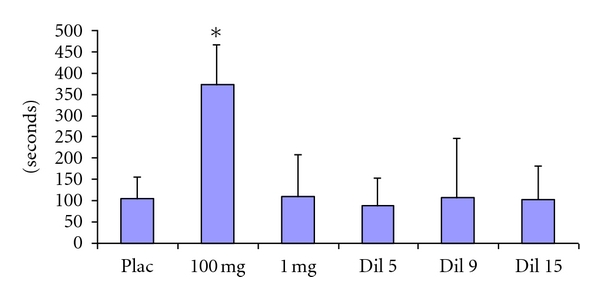
Induced Hemorrhagic Time (expressed in seconds). Effects of aspirin in 100 or 1 mg/kg or dilutions 5, 9, or 15 in normal mice. Data expressed as mean ± SD and analyzed with on-way ANOVA followed with Dunnet's multiple comparison test; *n* = 12/group.

**Figure 2:**
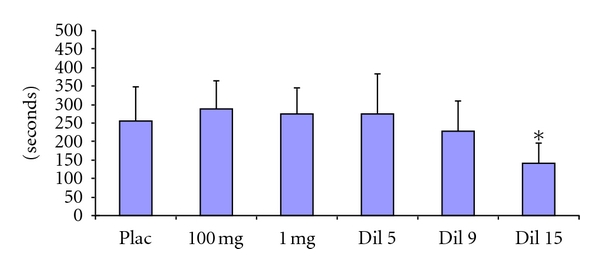
Induced Hemorrhagic Time (expressed in seconds). Effects of aspirin in 100 or 1 mg/kg or dilutions 5, 9, or 15 in COX 1 −/− mice. Data expressed as mean ± SD and analyzed with on-way ANOVA followed with Dunnet's multiple comparison test; *n* = 12/group.

**Figure 3:**
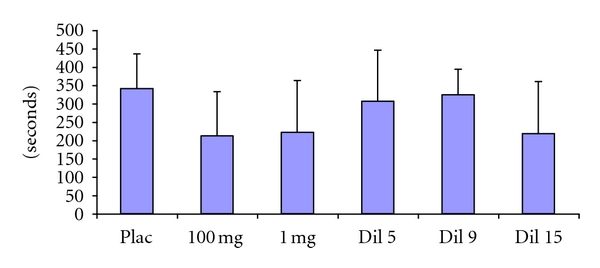
Induced Hemorrhagic Time (expressed in seconds). Effects of aspirin in 100 or 1 mg/kg or dilutions 5, 9, or 15 in COX 2 −/− mice. Data expressed as mean ± SD and analyzed with on-way ANOVA followed with Dunnet's multiple comparison test; *n* = 12/group.
